# Molecular Characteristics of Kraft-AQ Pulping Lignin Fractionated by Sequential Organic Solvent Extraction

**DOI:** 10.3390/ijms11082988

**Published:** 2010-08-16

**Authors:** Kun Wang, Feng Xu, Runcang Sun

**Affiliations:** 1 Institute of Biomass Chemistry and Technology, Beijing Forestry University, Beijing 100083, China; E-Mails: wangkun@bjfu.edu.cn (K.W.); xfx315@bjfu.edu.cn (F.X.); 2 State Key Laboratory of Pulp and Paper Engineering, South China University of Technology, Guangzhou 510640, China

**Keywords:** Kraft-AQ pulping lignin, characteristics, GPC, ^13^C-NMR

## Abstract

Kraft-AQ pulping lignin was sequentially fractionated by organic solvent extractions and the molecular properties of each fraction were characterized by chemical degradation, GPC, UV, FT-IR, ^13^C-NMR and thermal analysis. The average molecular weight and polydispersity of each lignin fraction increased with its hydrogen-bonding capacity (Hildebrand solubility parameter). In addition, the ratio of the non-condensed guaiacyl/syringyl units and the content of β-*O*-4 linkages increased with the increment of the lignin fractions extracted successively with hexane, diethylether, methylene chloride, methanol, and dioxane. Furthermore, the presence of the condensation reaction products was contributed to the higher thermal stability of the larger molecules.

## 1. Introduction

The global energy market provides about 370 exajoules of energy per year, which is equivalent to the energy consumption of about 170 million barrels of oil per day. Approximately 87% of the total energy purchase comes from fossil fuels [1]. This dependence on fossil fuels has increased the amount of carbon dioxide in the atmosphere by about one-third since the preindustrial era. It is now widely believed that the continuous dependence on fossil fuels is causing climate change, and that the consequences are both uncertain and unwelcome. Thus, sustainable and secure fuel supply from renewable sources such as forest and agricultural resources is generating interest with respect to electricity, heat, and transportation fuel [2].

Pulping paper is one of the most important applications from agricultural resources. The kraft pulping process is the most popular delignification process because of 1) its good recovery yield of pulping chemicals, 2) its suitability for all fiber materials, 3) its superior strength properties of Kraft pulps and 4) its high energy efficiency. During this process, the wood degradation results in a loss of about 35% of the carbohydrates due to the “peeling reaction”. In the 1970’s, anthraquinone (AQ) and its derivatives were initially investigated as pulping additives [3]. The addition of small amounts of AQ (0.04%–0.1%) accelerates the delignification rate, reduces the time required for the carbohydrate peeling reaction [4,5], and protects carbohydrates, as a result of which the pulp yield is significantly elevated [6]. Lignin is the main component in black liquid after pulping, and was traditionally viewed as a waste material or a low value byproduct [7,8]. Recently, lignin, as the most abundant natural aromatic material, is being considered for new economical applications such as bio-fuel, binder, dispersant or emulsifier, phenolic resins, carbon fibers [9,10], automotive brakes, wood panel products [11], polyurethane foams [12], epoxy resins for printed circuit boards [13] and so on. The lack of commercial high-value applications of lignin is mainly due to its heterogeneous molecular structure. Unlike most natural polymers, lignin molecules are extremely complicated due to their natural variability. Characteristic analysis could facilitate the specific application, allow the reliable monitoring of modification process, and clear the relationship between properties and performance. Generally, it is considered that lignin molecules consist of three *p*-hydroxycinnamyl alcohol precursors: *p*-coumaryl, coniferyl and sinapyl alcohols. Several interunit linkages including β-*O*-4, α-*O*-4, β-5, β-β, 4-*O*-5, 5–5 are formed by dehydrogenation, cross-coupling, and dehydrodimerization reactions during the biosysthesis process of macromolecular lignin. Thereby, lignin is subjected to the separation process prior to the molecular characterization for the sake of studying the molecular and structural heterogeneity.

In the present investigation, the Kraft-AQ lignin recovered from the black liquor was fractionated according to its hydrogen-bonding capacity (Hildebrand solubility parameter) in different organic solvents. The molecular characteristics of each obtained fraction in terms of chemical component, molecular weight distributions, UV, FT-IR, ^13^C NMR and thermal stability, were discussed. The results offer a better understanding of the structural complexity of lignin molecule and further utilization.

## 2. Results and Discussion

### 2.1. Yield and Molecular Weight Distribution

**Figure 1:**
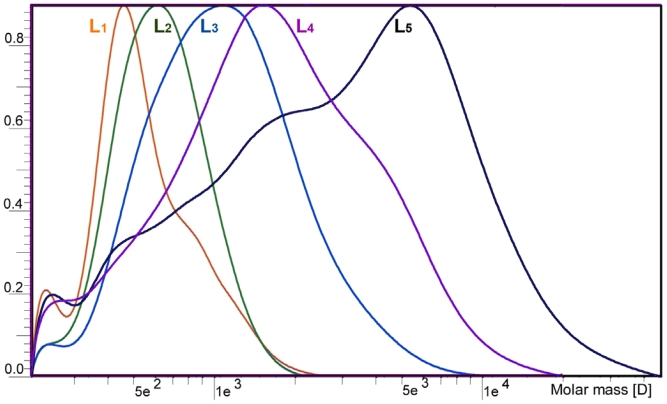
Molecular weight distributions for lignin fractions F_1_, F_2_, F_3_, F_4_ and F_5_.

Data shows in Table 1, yields of F_1_ (hexane) and F_2_ (diethylether) were only 0.2% and 1.6% of the starting lignin. Methylene chloride and methanol, as strong hydrogen-bonding capacity solvents, dissolved large portions of the initial lignin, and yielded 33.4% for F_3_ (methylene chloride) and 59.1% for F_4_ (methanol), respectively. Dioxane completely solubilizes the residual lignin (4.4% for F_5_). The molecular weight distributions of the five lignin fractions are shown in Figure 1. The weight-average(—*,M*_w_) and number-average (—*,M*_n_) molecular weights as well as the polydispersity (—*,M*_w_/—*,M*_n_) of all lignin fractions are listed in Table 1. A steady increment of the molecular weight was observed from F_1_ (—*,M*_w_ = 493 g/mol) to F_4_ (—*,M*_w_ = 2468 g/mol). It is suggested that the solvents used in the present work were selective for separating Kraft-AQ lignin from the low molecular weight to the high molecular weight, depending on the increasing Hildebrand solubility parameters from σ(hexane) = 7.3 (cal/cm^3^)^1/2^ to σ(methanol) = 14.5 (cal/cm^3^)^1/2^. Similarly, the polydispersities of the first four lignin fractions were (F_1_–F_4_) also elevated from 1.0 to 1.5 with the stronger hydrogenbonding capacity-solvents. Mörck *et al*. [15,16] also reported similar results in the study of the Kraft lignin from both hardwood and softwood by successive extraction with organic solvents. Due to the complete solubilization of lignin in dioxane, F_5_ (—*,M*_w_ = 13,651 g/mol) contained a small amount of material with extremely high molecular weight and polydispersity (4.5%). As shown in the molecular weight distribution curves, the distinctive peak obviously shifted to the higher molecular weight region and became broader from F_1_ to F_5_, indicating a gradual increase of the molecular weight and polydisperisty. As the profile of F_5_, the “tail” of the very high molecular weight material extended to above 50,000 g/mol.

### 2.2. Composition of Phenolic Acids and Aldehydes

Alkaline nitrobenzene oxidation has been widely used for assaying and identifying the structural units of lignin. In the case of oxidation, the three constitutive monomeric lignin units *p*-hydroxyphenyl, guaiacyl, and syringyl produce the corresponding *p*-hydroxybenzaldehyde, vanillin, and syringaldehyde. Table 2 shows the characterization of phenolic acids and aldehydes in the molecules of each fraction. The standard deviations are between 6% and 15%. Aldehydes were found to be the predominant oxidation products. The presence of *p*-hydroxybezonic acid and *p*-hydroxybenzaldehyde, which might have been derived from non-condensed *p*-hydroxyphenyl units, indicates the incorporation of *p*-hydroxycinnamoyl alcohol in the Kraft-AQ pulping lignin. The syringl/guaiacyl ratio is an important criterion for assessing the degree of condensation of lignin. Data shows the S/G value gradually decreased with increasing fraction number from 1.58/1 in F_1_ to 1.40/1 in F_5_. Similar trends were also found in the study of lignin fractions obtained by the separation with organic solvents [15–17], but contradicted the results obtained by Thring *et al*. [18] in the study of ALCELL^®^ lignin. Vander Klashorst and Strauss [17] attributed the decreasing S/G value to the following reasons: (1) The monomethoxyphenyl rings can be bonded to the lignin macromolecule at the 1- and 5- ring positions, but syringyl (3,5-dimethoxyphenyl) rings cannot form the 5-linkage; (2) The 4-hydroxy-3-methoxy substituted phenyl groups have a reactive site on the phenyl ring to participate in the condensation reactions. The guaiacyl compounds can therefore be expected to condense forming higher molecular weight compounds. The different conclusions obtained by Thring and co-workers were probably due to the fact that the β-aryl ethers were slightly degraded during the mild organosolv pulping [16].

### 2.3. UV Analysis

**Figure 2:**
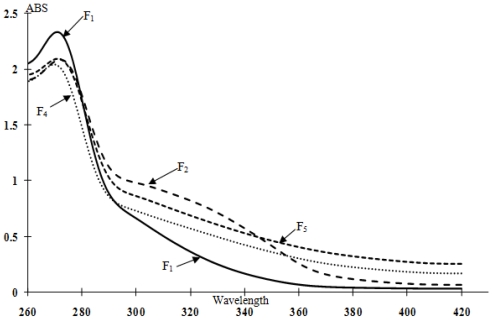
UV spectra of lignin fractions F_1_, F_2_, F_3_ *^a^*, F_4_ and F_5_. *^a^* The UV spectrum of F_3_ was totally overlapped with that of F_2_.

UV spectroscopy was used to semiquantitatively determine the purity of lignin or monitor the lignin distribution among various tissues of gymnosperm and dicotyledonous angiosperm with respect to concentration [19]. In this study, UV absorption measurements of the five lignin fractions were carried out in dioxane/water mixture, which solubilizes the lignin but limits the detectable wavelength ranged to 260–420 nm [20]. As shown in Figure 2, the spectra of all lignin fractions illustrate the basic style of lignin, which have a maximum absorption coefficient at about 270 nm, originating from phenolic hydroxyls conjugated to α-carbonyl, carbon-carbon double bonds, or biphenyl groups in lignin. The absorption at 320 nm in the spectra of F_2_ and F_3_ is undoubtedly due to the small quantity of the associated hydroxycinnamic acids (*p*-coumaric and ferulic acids). Furthermore, the increment of the relative absorption co-efficient from F_5_ to F_1_ revealed that the relatively higher purity induced by the organic solvents extraction process.

### 2.4. FT-IR Spectra Analysis

**Figure 3:**
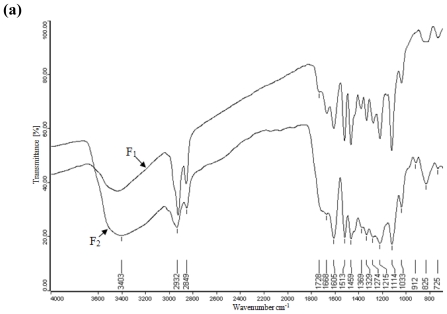

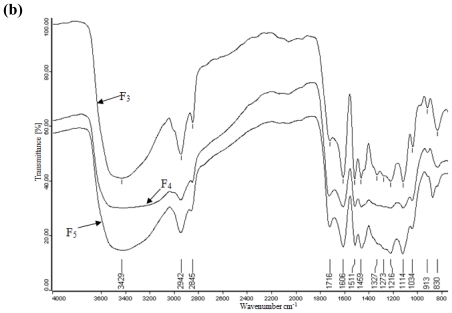
FT-IR spectra of lignin fractions **(a)** F_1_ and F_2_, **(b)** F_3_, F_4_ and F_5_.

FT-IR spectroscopy, which is a complementary and extensively used method, yields information about the molecular conformation and hydrogen bonding patterns. As shown in the spectra (Figure 3(a) and (b)), the wide absorption at 3403 (3429, data in Figure 3 (b)) cm^−1^ is attributed to O-H and C-H stretching, corresponding to the aliphatic moieties in the lignin. The bands at 2932 (2942) cm^−1^ and 2849 (2845) cm^−1^ arise from the C-H stretching of methyl, methylene and methine groups respectively. The absorptions at 1605 (1606) cm^−1^ and 1513 (1511) cm^−1^ indicate the aromatic skeleton vibrations, while the band at 1459 (1459) cm^−1^ is assigned to the C-H deformations and aromatic ring vibrations. The sharp band at 1114 (1114) cm^−1^ and the shoulder at 1033 (1034) cm^−1^ represents the aromatic C-H in-plane deformation in syringyl type and guaiacyl type, respectively [21]. However, a slight difference can be clearly distinguished on closer examination. The shoulder at 1726 cm^−1^ in the spectrum F_2_ is undoubtedly assigned to the ester linkage of carboxylic groups in ferulic and *p*-coumaric acids as analyzed in the UV spectroscopy [22]. The presence of the band at 1716 cm^−1^ in the spectra F_3_, F_4_ and F_5_, which corresponds to the unconjugated ketone and carboxyl groups stretching, indicated that the unconjugated carbonyl groups were mainly distributed in the high molecular weight fractions and could not be easily fractionated by low σ value solvents. Conversely, the conjugated carboxyl absorption at 1668 cm^−1^ in the spectrum F_1_, which appeared as a small shoulder in F_2_ and totally disappeared in the spectra F_3_, F_4_ and F_5_, manifested that the lignin fractions with conjugated carbonyl groups were liable to dissolve in hexane and diethylether, both of which have lower Hildebrand solubility parameters compared to other organic solvents used in this report. Moreover, the band at 1369 cm^−1^ for the non-etherified phenolic -OH groups, mainly resulting from the cleavage of β-*O*-4 and α-*O*-4 linkages, only presented in the spectra F_1_ and F_2_. Under alkaline pulping conditions, severe condensation reactions accompanied the cleavage of ether linkages in phenolic phenylpropane units [17]. The non-etherified phenolic -OH groups, therefore, mainly occurred in the low molecular weight fractions and took a small part of total Kraft-AQ lignin.

### 2.5. Thermal Analysis

**Figure 4:**
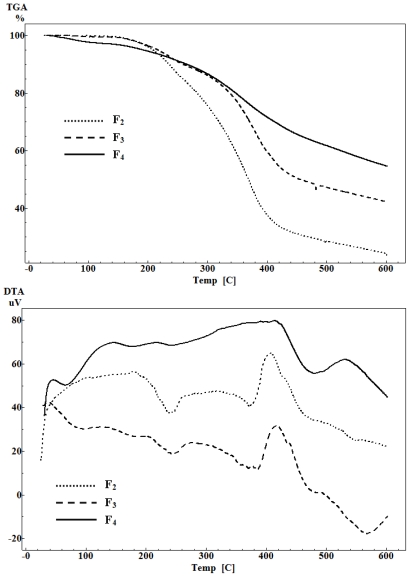
TGA/DTA curves of lignin fractions F_2_, F_3_ and F_4_.

The thermal degradation patterns of the lignin fractions F_2_, F_3_ and F_4_ are shown in Figure 4, giving evidence of their thermal stability. Among the lignocellulosic materials, lignin is the most thermo-stable component mainly due to the inherent structure of aromatic rings with various branches. Because the reactivity range of lignin is quite wide, the degradation of lignin occurs within a wide temperature range. According to previous reports [23–26], the thermal degradation of lignin could take place in the following steps: 1) cleavage of α- and β-aryl-alkyl-ether linkages occurs between 150 °C and 300 °C; 2) aliphatic side chains start splitting off from the aromatic ring around 300 °C; 3) the carbon-carbon linkage between lignin structural units is cleaved at 370–400 °C [23]; decomposition or condensation of aromatic rings is believed to take place at 400–600 °C [24]. Most lignin show their maximum rate of weight loss between 300 °C and 400 °C [25,26]. As illustrated in Figure 6, about 5% losse in the weight of F_4_ were ascribed to the evaporation of the absorbed water heating from room temperature to 150 °C. On the whole, the onset of thermal degradation temperatures (180 °C for F_2_, 196 °C for F_3_, and 208 °C for F_4_), the non-volatile residues (23% for F_2_, 43% for F_3_, and 55% for F_4_), and the temperature of maximum weight loss rate (342 °C for F_2_, 377 °C for F_3_, and 392 °C for F_4_) were increased with the increasing fraction number. This indicated the higher thermal stability with the greater molecular weights. Even though the degradation and fragmentation reactions are major reactions of the Kraft process, a variety of condensation and other reactions occur and are believed to contribute to the formation of the more heterogeneous structure and stable carbon-carbon bonds, which result in the low reactivity of residual lignin. DTA measures the amount of heat liberated or absorbed by the sample when phase transition occurs (such as melting or vaporization) or when it undergoes any chemical reaction. This heat is determined by measuring the temperature difference between the sample and an inert reference. As can be seen from Figure 4, the exothermic peak at around 400 °C obviously revealed a shift towards the higher temperature from F_2_, to F_3_, and to F_4_. It is further confirmed by the increasing trend of thermal stability of the fractionated lignin fractions with higher molecular weights.

### 2.6. ^13^C NMR Spectra Analysis

**Figure 5:**
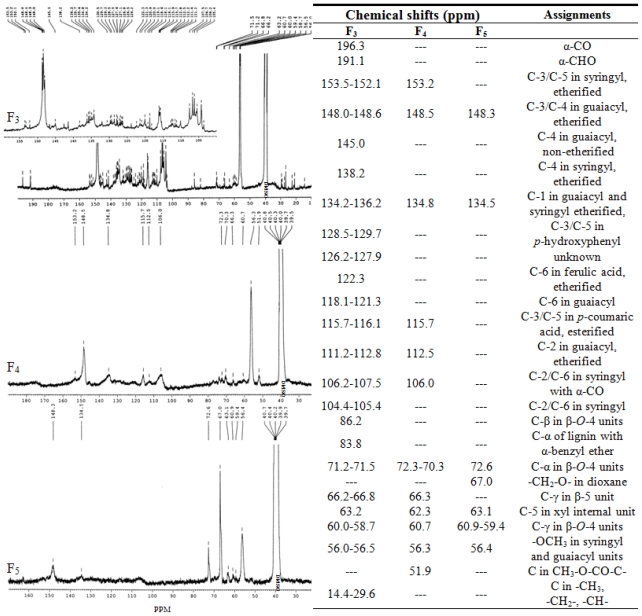
^13^C NMR spectra and peak assignments of lignin fractions F_3_, F_4_ and F_5_.

Figure 5 illustrates the ^13^C NMR spectra of the lignin fractions F_3_, F_4_ and F_5_. The peak assignment in Table 3 is based on the comparison with the data reported by Mörck *et al*. [15,16], van der Klashorst and Strauss [17], Sun *et al*. [27], and Holtman *et al*. [28]. The signals for aromatic part of the lignin appear in the region between 104 and 160 ppm. The presence of the *p*-hydroxyphenyl unit signals indicates that the Kraft-AQ lignin obtained from *Eucalyptus pellita* can be categorized into S-G-H lignin. Lapierre *et al*. [29] compared the integrated areas under the peak of C-2/C-6 syringyl (around 106 ppm) to that of C-2 guaiacyl (around 111 ppm) in order to quantify the syringyl/guaiacyl ratio. In Figure 5, the syringyl/guaiacyl ratio decreased from F_3_ to F_4_, and to F_5_, which is in accordance with the results from the determination of alkaline nitrobenzene oxidation. Signals in the 86–60 ppm region are attributed to oxygenated and non-oxygenated interunit linkages in lignin. As observed, the intensity of the signals, which assigned to the C_γ_, C_β_ and C_α_ in the β-*O*-4 ether bonds, increased in the larger molecule (F_5_). This result indicates that most of the residual β-*O*-4 linkages locate in the “core” macromolecule, which was relatively protected during the pulping process. This result further confirmed the absence of the absorption at 1369 cm^−1^ for non-etherified phenolic -OH groups in the FT-IR spectra. A similar trend was previously reported by Mörck *et al*. [15]. They fractionated Kraft lignin by the organic solvent extraction (dichloromethane, n-propyl alcohol, methanol, and methanol/dichloromethane: 70/30), and found no traces of signals from β-*O*-4 structure in the ^13^C NMR spectrum of the fraction dissolved in CH_2_Cl_2_.

In the F_3_ spectrum, two weak signals were observed. One is attributed to C-5 in xyl internal unit (62.3 ppm) and the other is assignd to C_α_ in lignin moieties with an α-benzyl ether linkage to polysaccharides (83.8 ppm). It is implied that d-xylose is probably associated with lignin through a α-benzyl ether bond. The presence of an α-benzyl ether linkage between the lignin and polysaccharides was also observed by Xie and Terashima [30] in the study of rice straw lignin traced by ^13^C NMR spectroscopy. Additionally, in the spectrum of F_3_, signals at 112.2–112.8 and 112.3 ppm demonstrated noticeable amounts of esterified *p*-coumaric and ferulic acid in the Kraft-AQ pulping lignin. Sun *et al*. [31] reported that *p*-coumaric acid links to lignin by ester bonds, while ferulic acid links to lignin by ether or ester bonds. Furthermore, another striking characteristic of the F_3_ ^13^C NMR spectrum is the peaks in the 14.4–29.6 ppm region, which is assigned to the saturated hydrocarbon structures in the aliphatic side-chains. In the Kraft cooking process, methine-, methylene-, and methyl groups are formed in the aliphatic side-chains of lignin from degradation of phenolic β-aryl ethers or condensation with formaldehyde. However, it must be noted that these saturated structures may partly arise from fatty acids or other non-volatile extractives since the starting material used in the pulping process was not extractive-free [26].

## 3. Experimental Section

### 3.1. Kraft-AQ Pulping

The black liquor was supplied by the pulping and papermaking laboratory of Beijing Forestry University. The Kraft-AQ pulping of *Eucalyptus pellita* was performed in a stainless steel batch reactor using 17% w/v Na_2_O, 20% w/v Na_2_S, and 0.1% w/v AQ in a liquor/wood ratio of 4.5/L (g/g). The initial temperature was 80 °C, and heated at the rate of 0.9 °C/min to reach the final temperature 170 °C and kept for 110 min. Afterward, the reaction medium was cooled and filtered in a 100% polyester cloth to separate the black liquor.

### 3.2. Lignin Recovery from Black Liquor

A 100 mL volume of the black liquor was treated with 6 M aqueous HCl and vigorous stirring (at room temperature) to obtain pH 2.0. After acidification, the precipitated lignin was centrifuged at 4000 rpm for 10 min, and then thoroughly washed with acidified water (pH 2.0), oven-dried at 60 °C for 16 h until constant mass and stored in a desiccator. It was reported that the further purification process by redissolving and precipitating could not only remove the extraneous organic contaminants, but also probably reduce the low molecular weight fractions [32]. Therefore, in this study, the Kraft- AQ lignin was only washed with acidified water (pH 2.0) several times in order to maintain the whole molecular weight distribution. The main impurity, the content of carbohydrate, was determined to be less than 1% according to the Chinese standard methods in the papermaking industry (GB/T 2677). Xylose accounted for about 70% of the total monosaccharide.

### 3.3. Separation Process

**Figure 6:**
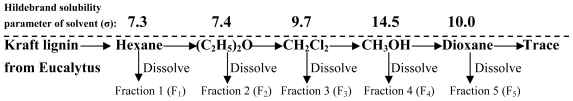
Scheme for the separation process of lignin from black liquor of *Eucalyptus pellita* Kraft-AQ pulping.

The Kraft-AQ lignin was fractionated according to the solubility in different organic solvents. The solvent selection was based on the result of the solubility tests conducted on ALCELL^®^ lignin by Thring *et al*. [18], and in the order of hexane (σ = 7.3), diethylether (σ = 7.4), methylene chloride (σ = 9.7), methanol (σ = 14.5), and dioxane (σ = 10.0), where σ (cal/cm^3^)^1/2^ represents the Hildebrand solubility parameter. Schuerch [33] discovered that the lower molecular weight lignin was soluble in the organic solvents with weak or moderate hydrogen-bonding capacity. The scheme of the separation process is illustrated in Figure 6. Dioxane was placed last due to its complete solubilization of lignin, even though its Hildebrand solubility parameter is less than that of methanol [18]. In this experiment, 5.0 g Kraft-AQ lignin was used as starting material. In each step the lignin was fractionated in a Soxhlet extractor, and each fraction was considered to be sufficiently removed by the very light colored supernatant. The supernatant liquid was evaporated under reduced pressure, and then ovendried at 60 °C for 16 h. Each fraction was stored in a desiccator for further analysis. The deviations of these contents from their respective means were all less than 5%. All chemicals were analytical grade.

### 3.4. Analytical Method

The chemical composition of phenolics was measured by alkaline nitrobenzene oxidation method, which was performed at 145 °C for 2.5 h, and determined by high-performance liquid chromatography (HPLC) on a ZORBAX Eclipse XDB-C_18_ HPLC column of dimensions 250 × 4.6 mm (1200 series, Agilent Technologies, USA). The identification of the individual compound was detected at 280 nm UV by computer comparison of the retention times and peak areas with the authentic phenolics. The molecular-average weights of the lignin fractions were determined by gel permeation chromatography (GPC) on a PLgel 5 m Mixed-D column. Because derivatization could increase the average molecular weights of lignins [34], the sample was simply dissolved in tetrahydrofuran (0.2% w/v) and a 20 μL solution was injected. The column was operated at 30 °C and eluted with tetrahydrofuran at a flow rate of 0.5 mL/min. To calibrate the column, monodisperse polystyrene of the known molecular weight was used as the standard.

UV spectra were obtained using a Techcomp 2300 diode array spectrophotometer. A lignin sample (5 mg) was dissolved in 10 mL of dioxane–water (95%, v/v). 2 mL aliquot was diluted to 10 mL with 50% (v/v) dioxane–water, and the absorbance between 260 and 420 nm was measured. FT-IR spectra were recorded by an FT-IR spectrophotometer (Tensor 27, Bruker, Germany) using a KBr disc containing 1% finely ground sample. The IR absorption was measured within the range 4000 to 400 cm^−1^ with an accumulation of 32 scans with a resolution of 2 cm^−1^. The ^13^C-NMR spectra of the lignin fractions were obtained using a Bruker MSL-300 (Germany) spectrometer (74.5 MHz). Sample concentrations were approximately 250 mg in 1.0 mL DMSO-*d*_6_, and placed in 5 mm i.d. NMR tubes and spun at a temperature of 25 °C for 18 h. Acquisition time was 1.2 s and pulse was 80° (7 μs). The instrument was set to proton inverse gated decoupled mode and the delay between scans was 1.2 s. Thermal analysis of the lignin fractions was performed using thermo gravimetric analysis (TGA) and differential thermal analysis (DTA) on a simultaneous thermal analyzer (DTG-60, Shimadzu, Japan). The sample was heated from room temperature to 600 °C at a rate of 10 °C/min with a nitrogen flow of 30 mL/min.

## 4. Conclusions

In summary, the Kraft-AQ lignin was fractionated by a series of extractions using organic solvents (hexane, diethylether, methylene chloride, methanol and dioxane) with an increasing hydrogen-bonding capacity. The separation process resulted in five fractions containing 0.2, 1.6, 33.4, 59.1, and 4.4% of the total lignin, respectively. The average molecular weight increased from 493 g/mol of F_1_ to 2468 g/mol of F_4_, reflecting an increasing degree of branching and condensation. The high molecular weight (13651 g/mol) and polydispersity (4.5) of F_5_ were probably due to the “tail” of the very high molecular weight material shown in the molecular weight distribution curve (Figure 2). The molar ratio of non-condensed syringyl/guayacyl units decreased gradually from F_1_ (1.58) to F_5_ (1.40), probably due to the higher reactive activity of monomethoxyphenol rings. From ^13^C NMR analysis, β-*O*-4 ether linkage was substantially cleaved during the Kraft-AQ pulping process, and the content of β-*O*-4 structure was higher in the higher molecular weight fraction. Additionally, the thermal stability also increased with the average molecular weight.

## Figures and Tables

**Table 1:** Yields (% dry matter, w/w), weight-average (—*,M*_w_) and number average (—*,M*_n_) molecular weights and polydisperisty (—*,M*_w_/—*,M*_n_) of the lignin fractions.

	Lignin fractions^a^				
	F_1_	F_2_	F_3_	F_4_	F_5_
Yield^b^^c^	0.2	1.6	33.4	59.1	4.4
—*,M*_w_	493	686	1174	2468	13651
—*,M*_n_	492	665	1012	1675	3053
—*,M*_w_/—*,M*_n_	1.0	1.0	1.2	1.5	4.5

a F_1_, F_2_, F_3_, F_4_, and F_5_ represent the lignin fractions fractionated from *Eucalyptus pellita* Kraft-AQ pulping lignin by hexane, ethylether, methylene chloride, methanol, and dioxane, respectively.

b % dry matter of the fractionated lignin.

c The standard deviation is less than 2%.

**Table 2:** The content of phenolic acids and aldehydes (% relative) obtained by alkaline nitrobenzene oxidation of each lignin fractions.

Phenolic acids and aldehydes	Lignin fractions^a^				

	F_1_	F_2_	F_3_	F_4_	F_5_
Vanillic acid	5.7	6.5	7.8	5.4	9.1
Vanillin	21.1	23.1	23.8	24.9	25.9
Acetovanillone	6.0	6.2	8.2	9.3	6.3
Syringic acid	2.9	10.4	11.6	15.8	22.9
Syringaldehyde	28.9	29.1	30.1	28.2	25.3
Acetosyringone	20.0	16.6	16.5	13.2	9.8
*p*-Hydroxybezonic acid	15.0	7.6	1.7	2.8	ND^b^
*p*-Hydroxybenzaldehyde	0.4	0.5	0.3	0.4	0.7
S/G^c^	1.58	1.57	1.46	1.44	1.40

a Corresponding to the lignin fractions in Table 1.

b Not detectable.

c G represents the total moles of vanillin, vanillic acid, and acetovanillone and S represents the total moles of syringaldehyde, syringic acid and acetosyringone.
